# Changes in nitrogen metabolism and antioxidant enzyme activities of maize tassel in black soils region of northeast China

**DOI:** 10.3389/fpls.2014.00515

**Published:** 2014-10-02

**Authors:** Hongwen Xu, Yan Lu, Zhiming Xie, Fengbin Song

**Affiliations:** ^1^School of Urban and Environmental Science, Huaiyin Normal UniversityHuaian, China; ^2^Northeast Institute of Geography and Agroecology, Chinese Academy of SciencesChangchun, China

**Keywords:** maize, tassel, nitrogen metabolism, antioxidant enzyme, black soils, China

## Abstract

Two varieties of maize (*Zea mays* L.) grown in fields in black soils of northeast China were tested to study the dynamic changes of nitrogen metabolism and antioxidant enzyme activity in tassels of maize. Results showed that antioxidant enzyme activity in tassels of maize increased first and then decreased with the growing of maize, and reached peak value at shedding period. Pattern of proline was consistent with antioxidant enzyme activity, showing that osmotic adjustment could protect many enzymes, which are important for cell metabolism. Continuous reduction of soluble protein content along with the growing of maize was observed in the study, which indicated that quantitative material and energy were provided for pollen formation. Besides, another major cause was that a large proportion of nitrogen was used for the composition of structural protein. Nitrate nitrogen concentrations of tassels were more variable than ammonium nitrogen, which showed that nitrate nitrogen was the favored nitrogen source for maize.

## INTRODUCTION

Maize (*Zea mays* L.) pollination is one of the most important and complicated phases of crop development, pollination in maize occurs only if pollen shed by the tassel is captured by the stigmas (silks) on the ear (Kaleita et al., 2006). Nitrogen is referred to be the most important mineral nutrient, whose availability is most likely to limit plant growth. Among these important plant nutrients, nitrogen is known as an important element to synthesize the essential cellular components for the processes of pollination and fertilization. Nitrogen is one of the most critical components of amino acids, nucleic acids, coenzymes, and other plant metabolites (Andrews et al., 2007; Varisi et al., 2008), its deficiency can result in the abnormal pollen grain. As the major nitrogenous compounds in plants, amino acids play key roles in supplying substrates for different kinds of cellular metabolism, such as energy generation and cell wall synthesis, which are imperative to the growth and developmental processes of plants (Azevedo et al., 2006; Ferreira et al., 2006). Further study is needed on the role of nitrogen metabolism in agricultural research, because it involves in pollen grain formation and quality improvement. Nitrate and ammonium nitrogen as principal nitrogen sources play essential roles in various physiological and metabolic functions in plants. Nitrate can be absorbed by plant roots and then be converted to ammonium by the sequential reductive action of the enzymes (Yang et al., 2013).

Reactive oxygen species (ROS) can be continually produced as natural products accompanied by nitrogen metabolic processes in plants, and excessive ROS will degrade polyunsaturated lipids, forming malondialdehyde (MDA; Mittler, 2002). MDA is always looked upon as the natural product and a reflection of the degree of membrane lipid peroxidation. Catalase (CAT) and peroxidase (POD) are considered to be the major antioxidant enzyme for scavenging ROS, and the scavenging ability is associated with antioxidant enzyme activities in plants (Xu et al., 2011). Plant could regulate osmotic potential through the accumulation of osmotic adjustment substance (Kishor et al., 2005; Sharma and Dietz, 2006). As one of the important osmotic adjustment substances in plants, proline plays important roles in improving cell membrane integrity by regulating the hydration among protein molecules, and protecting enzymes from injury through some degree of passivation (Liu et al., 2006). This is a very effective self-protecting mechanism, which can not only protect cells from injury but also maintain original biological processes.

Extensive researches on nitrogen metabolism in maize have been documented, however, most are about vegetative organ (Pommel et al., 2006; Kim and Woloshuk, 2008; Wang et al., 2009; Wei et al., 2010), and there are limited systematic investigations on reproductive organs (Xu et al., 2012), moreover, there is no report on nitrogen metabolism as well as antioxidant enzyme activities in tassels of maize. Studying in the nitrogen metabolism is particularly important for further insight into physiological and biochemical mechanisms for maize pollination. In order to analyze changes of nitrogen metabolism and relationships with antioxidant enzyme activities as well as to understand the nitrogen metabolic characteristic in tassels of maize. In this study, ZD 958 (compact type maize) and ND 364 (loosely type maize) were selected as materials, which are widely cultivated in black soils region of northeast China were chosen in the present experiment. Three major objectives were included in our study: (i) to study the changes of antioxidant enzyme activity and lipid peroxidation of tassels along with the growth of maize; (ii) to analyze changes of nitrogen metabolites during the whole growth period; (iii) to reveal the relationship between antioxidant enzyme and nitrogen metabolism in tassels of maize.

## MATERIALS AND METHODS

### EXPERIMENTAL DESIGN

The research was conducted at Agricultural Experimental Station (44°> 12′ N, 125°> 33′ E) of Northeast Institute of Geography and Agroecology. The basic characteristics of the soil (0–20 cm) are that it contains organic matter 26.9 g⋅kg^-1^, available nitrogen 118.8 mg⋅kg^-1^, available phosphorus 18 mg⋅kg^-1^, and available potassium 111 mg⋅kg^-1^; pH is 6.6. Two maize varieties, ZD 958 and ND 364 were adopted in this experiment. The experiment was arranged in two blocks, and each variety was cultivated in a block with an area of 1500⋅m^2^. Samples were collected in an “S” shape in each block. The study was randomly conducted in five sample plots with three replicates. The seeds were planted with agricultural fertilizer containing 60 kg⋅ha^-1^ of N, P, and K. An additional side-dressing of 56 kg⋅ha^-1^ N as ammonium nitrate was applied.

### ESTIMATION OF SOLUBLE PROTEIN AND PROLINE

Contents of soluble protein estimations were performed according to the methods of Bradford (1976), and proline content was determined using the method of Zhang and Qu (2003). 0.5 g of fresh tassel samples were extracted with 5 mL 3% sulfosalicylic acid, then put in boiling water for 10 min bath. 2 mL of extract after filtration was added to 6 mL assay medium including 2 mL 2.5% ninhydrin solution and 2 mL 17.5 M acetic acid, cultivated for 30 min at 100°>C, and then cooled. The colored product was extracted with 4 mL toluene with shaking, then the absorbance of the organic layer was measured at 520 n.

### DETERMINATION OF NITRATE AND AMMONIUM NITROGEN

Nitrate and ammonium concentration were determined with the salicylic acid method described by Cataldo et al. (1975).

### ANTIOXIDANT ENZYME ACTIVITIES DETERMINATION

Tassels were homogenized in 5 mL phosphate buffer at pH 7.8 containing 1 mM ethylene diamine tetraacetic acid, 1 mM dithiothreitol and 5 mL of 4% polyvinyl pyrrolidone and centrifuged at 10,000 × g for 20 min at 4°>C, and the supernatant was used to measure enzyme activity. CAT activity was measured according to Samantary (2002). 1 mL of the supernatant was added to the reaction mixture containing 1 mL of 0.1 M H_2_O_2_ and 3 mL of 0.1 sodium phosphate buffer. The reaction was discontinued by adding 10 mL at 2% H_2_SO_4_ after 1 min of incubation at 20°>C. The reaction mixture was then titrated against 0.01 M KMnO_4_ to determine the quantity of H_2_O_2_ used by the enzyme. Enzyme activity was expressed as mg H_2_O_2_ destroyed min^-1^ mg protein^-1^. POD activity was determined using guaiacol oxidation in a reaction mixture containing 50 mM phosphate buffer (pH 6.0), 20.1 mM H_2_O_2_, and enzyme extract. The increase in absorbance was recorded by the addition of H_2_O_2_ at 470 nm for 3 min (Bai et al., 1996). Malondialdehyde (MDA) was extracted with 10% trichloroacetic acid and determined at 450 nm, 532 nm and 600 nm following the procedures that were described by Dhindsa et al. (1981).

### MEMBRANE RELATIVE PERMEABILITY MEASUREMENTS

Tassel samples were washed with deionized water, followed by the introduction of small excisions and then incubated in deionized water. Membrane relative permeability was measured using a conductivity meter (DDS-11A, China).

### DATA ANALYSIS

The data was analyzed by one-way analysis of variance (ANOVA) followed by Duncan test at 0.05 level to compare the means using SPSS 16.0 for Windows.

## RESULTS

### SOLUBLE PROTEIN AND PROLINE CONTENT

**FIGURE 1:**
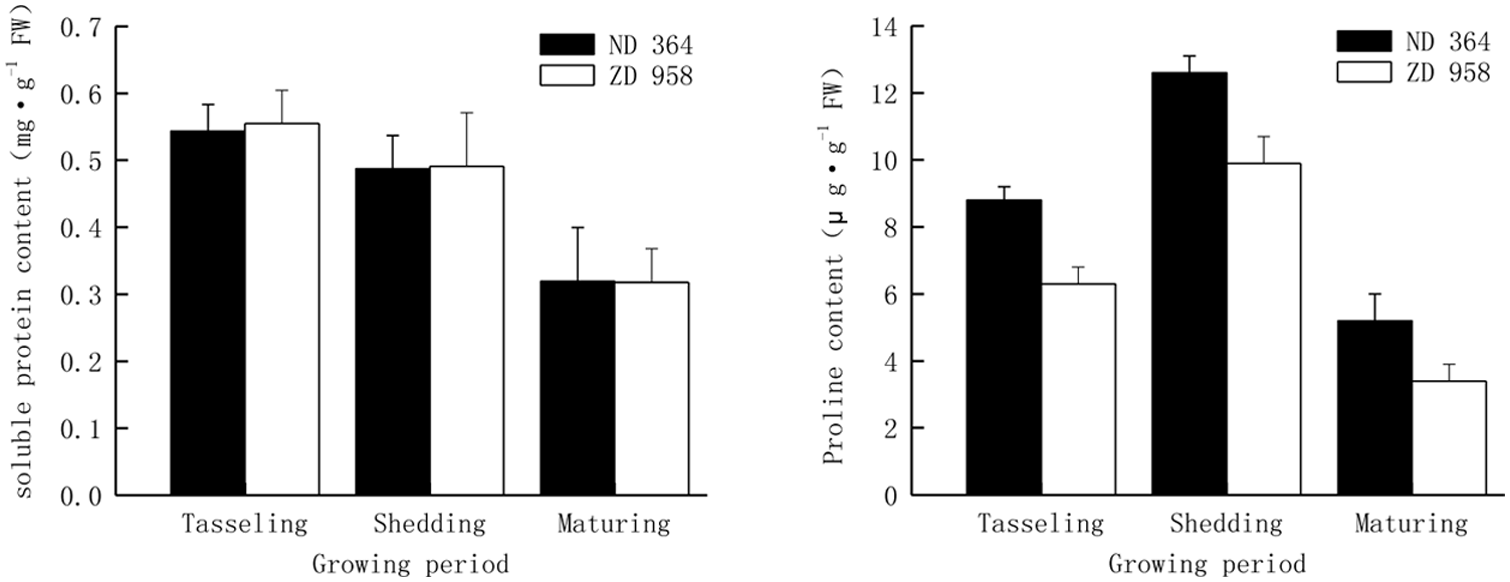
**Soluble protein and proline content in tassels of maize under field condition**.

Soluble protein and proline content were analyzed from tasseling to maturing period (**Figure 1**). Continuous reduction of soluble protein content in tassels along with the growing of maize for both varieties was obtained in the study. From tasseling to maturing period, soluble protein content decreased by approximately forty percent in two maize varieties. Contiguous decreasing range was found for both varieties, and there were no significant differences in diverse growth periods. It showed that there were the same changing tendencies in proline content in tassels of two maize varieties. Proline content in two maize varieties increased from tasseling period and achieved the highest level at shedding period, declined from their maxima and remained at the lower level at maturing period. Proline content in tassels of ND 364 was significantly higher than that in ZD 958.

### NITRATE AND AMMONIUM NITROGEN

**FIGURE 2:**
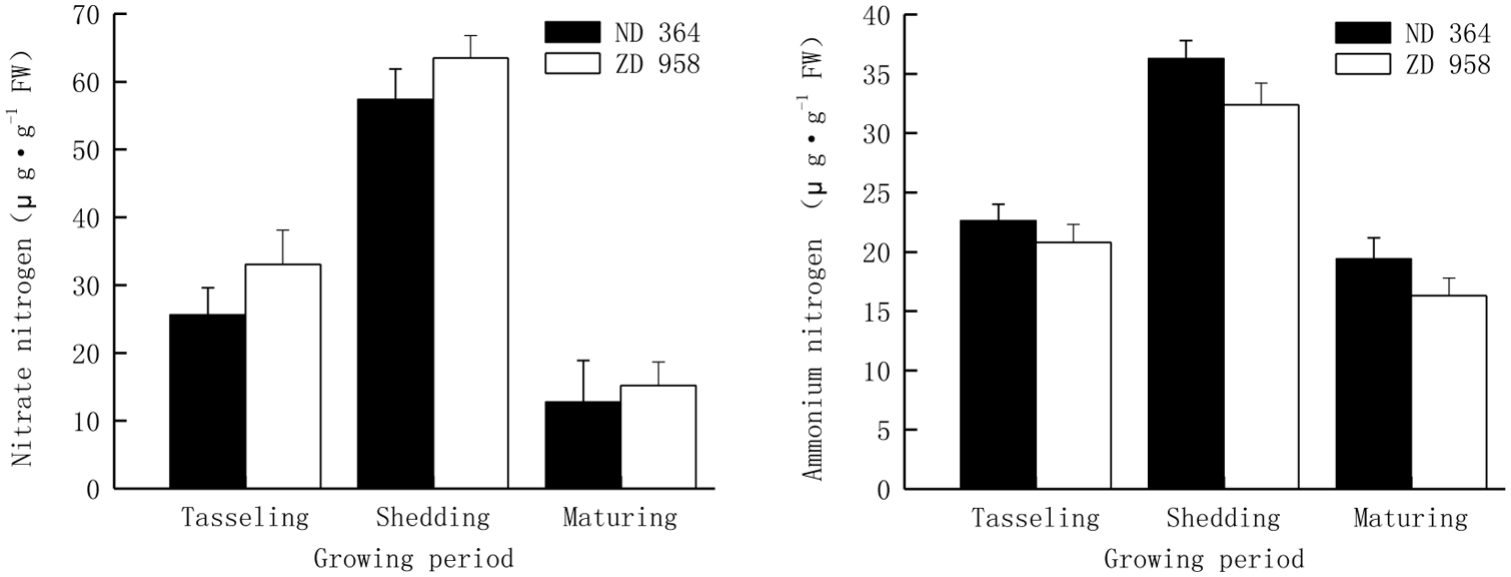
**Nitrate and ammonium nitrogen in tassels of maize under field condition**.

Nitrogen with different forms in soil, is one of the most important nutrients for maize growth, and the various forms of nitrogen play very important roles in determining the availability of nitrogen to plants. Plants have the ability to take up several chemical forms of nitrogen, the forms of nitrogen that most plants can use are nitrate (NO_3_^-^) or ammonium (NH_4_^+^). Thus, they are the two major important criteria for assimilation and reutilization of nitrogen. The similar tendency of contents of nitrate and ammonium nitrogen was found in tassels for both varieties, that is, it increased first and then decreased with the growing of maize, and reached the peak value at shedding period (**Figure 2**). Values of both nitrate and ammonium nitrogen decreased from shedding to maturing period, especially those of nitrate nitrogen sharply deceased by over 75% in two maize varieties.

### ANTIOXIDANT ENZYMES ACTIVITIES

**FIGURE 3:**
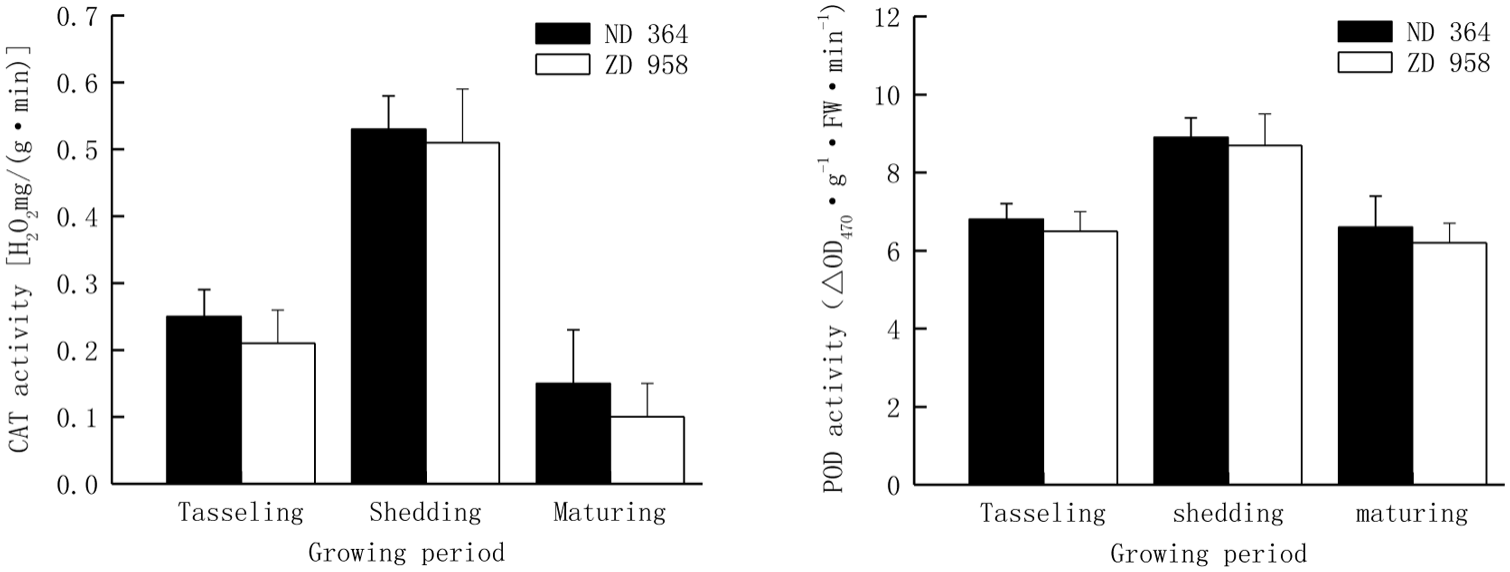
**CAT and POD activity in tassels of maize under field condition**.

The activities of antioxidant enzymes in tassels exhibited almost the same trend with the growing of maize and reached peak value at shedding period (**Figure 3**). The activities of CAT and POD decreased from shedding to maturing period, especially the CAT activity sharply reduced by over eight percent for both maize varieties, while the POD activity had a distinctly lower decrease in comparison with that of CAT in two maize varieties and retained a significantly higher value at maturing period. Both CAT activity and POD activity were significantly higher in tassels of ND 364 than those in ZD 958 during the whole growth stages.

### MEMBRANE RELATIVE PERMEABILITY AND LIPID PEROXIDATION

**FIGURE 4:**
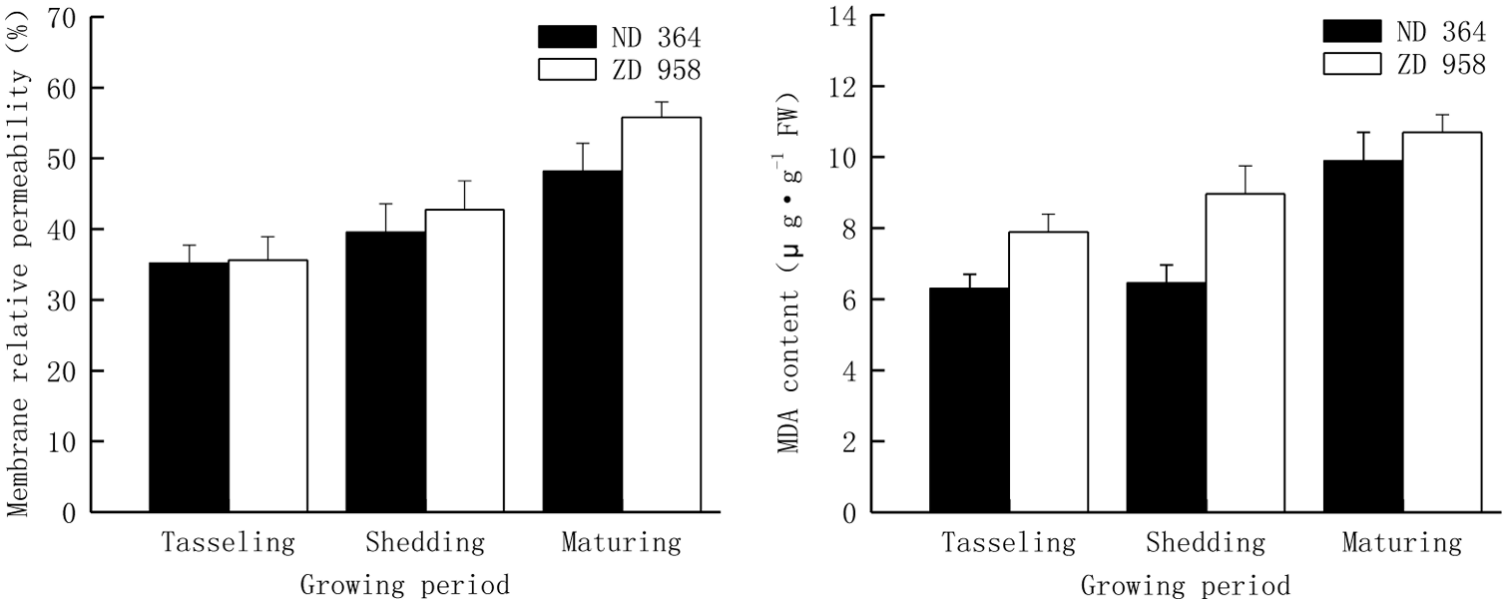
**Membrane relative permeability and MDA content in tassels of maize**.

Changes of membrane relative permeability and MDA content in tassels of two maize varieties displayed the same tendencies (**Figure 4**). Both membrane relative permeability and MDA content reached the highest values at maturing period and the lowest values at tasseling period. Both membrane relative permeability and MDA content in tassels were lower in ND 364 than those in ZD 958 during the whole growth stages. Compared with membrane relative permeability, the values of increased amplitude of MDA content were much higher.

Many empirical evidence has confirmed the relative permeability of membrane, and MDA may be involved in the activities of antioxidant enzymes. However, these relationships varied strongly with temporal and spatial variation. In this study, we examined two important antioxidant enzymes that affect photosynthesis. Regression analysis revealed a positively significant correlation between CAT and POD activities (**Table 1**). However, the changes of antioxidant enzymes in tassel of maize under field conditions had no relation to the relative permeability of membrane and MDA content. It could be inferred that the mechanism of an increase in activities of antioxidant enzymes seemed utterly different from membrane relative permeability and MDA content.

**Table 1:** Correlation coefficients between membrane relative permeability, MDA and activities of antioxidant enzymes.

Variety	Parameter	Membrane relative permeability	MDA	CAT	POD
ND364	Membrane relative permeability	1			
	MDA	0.9142*	1		
	CAT	0.1849	0.4565	1	
	POD	0.0693	0.2856	0.9686**	1

ZD958	Membrane relative permeability	1			
	MDA	0.9991**	1		
	CAT	0.1740	0.1520	1	
	POD	0.0754	0.0604	0.9771**	1

*P < 0.05, **P < 0.01.

## DISCUSSION

Intensive metabolism and dynamic changes of nitrogen had significant effects on quantity and quality of maize (Ren and Zhang, 2013; Tian et al., 2013), rational control of nitrogen nutrition was directly related to higher yield and quality (Liu et al., 2011; Wang et al., 2012; Mandal et al., 2013). Especially in the late growing periods, nitrogen metabolism was favorable for the differentiation of floret primordia (Guilpart et al., 2014). As one of the most important nitrogen-containing organic compounds, protein was vital for the growth of reproductive organs and sexual reproduction. Reduction in soluble protein content of tassels along with the growing of maize was due to quantitative material and energy provided for some new tissue formation needed in the course of pollen formation. And another major cause was that a large proportion of nitrogen was used for the composition of structural protein.

Nitrate being the source of nitrogen metabolism, the rate of nitrogen metabolism and nutrition requirements in tassel of maize can be reflected by nitrate utilization condition. Ammonium nitrogen is an important raw material to synthesize amino acids, however, its immense accumulation would be poisonous to plants. Study showed that the values of nitrate and ammonium nitrogen attained peak at shedding period, which means that sufficient material base for pollen grain formation could be provided by nitrogen. In the present investigation, more dramatic changes occurred in nitrate content compared with those in ammonium nitrogen, which suggested that nitrate was the preferred nitrogen source for maize. And a far higher proportion of ammonium nitrogen was found along with the growth periods as well, which could reduce the accumulation of nitrate effectively. However, the important roles of key enzymes for nitrogen metabolism were still obscure, which were needed to be further investigated.

It was estimated that huge amount of ROS were generated, such as superoxide anion radical, hydrogen peroxide and hydroxyl radical during the senescence of maize (Sharma et al., 2012; Hussain et al., 2013). High levels of ROS implied in lipid damage and alter membrane properties, which can lead to the increase of MDA content and membrane permeability (Song et al., 2013). However, plant can establish effective defense mechanisms against oxidative damage by induction of antioxidant enzymes (Weisany et al., 2012). CAT and POD are the important antioxidant enzymes, they play important roles in minimizing the adverse effects of ROS (Jaleel et al., 2009). After shedding period, CAT activities dropped swiftly, while POD activity decreased smoothly and remained at higher level in two maize varieties, which indicated that POD was the key ROS-scavenging enzyme. Present studies also found the tendency of antioxidant enzymes activity was opposed to MDA content after shedding period. MDA accumulating would inhibit the activities of the enzymes, on the contrary, reduction of enzymes activities could give rise to the accumulation of free radicals, thus increasing MDA content and plasma membrane through direct and indirect initiation of lipid peroxidation (Tang et al., 2010). Proline was shown to scavenge hydroxyl radicals and singlet oxygen, thus providing protection against ROS-induced cell damage (Yilmaz and Parlak, 2011). Change of proline was accorded with antioxidant enzymes, suggesting that osmotic adjustment could protect many enzymes that are important for nitrogen metabolism, all of which was conducive to the process of pollination and fertilization.

In conclusion, the consistencies of changes of antioxidant enzymes activities and proline content occurred in the tassels of maize, suggesting that osmotic adjustment could protect many enzymes which were important for nitrogen metabolism. Linear correlation coefficient indicated that the increase in activities of antioxidant enzymes were irrelevant to membrane relative permeability and MDA content. And our results also supported a speculation that POD was the key ROS-scavenging enzyme for its slow reduction of activities.

## Conflict of Interest Statement

The authors declare that the research was conducted in the absence of any commercial or financial relationships that could be construed as a potential conflict of interest.
